# The hybrid bio-robotic swarm as a powerful tool for collective motion research: a perspective

**DOI:** 10.3389/fnbot.2023.1215085

**Published:** 2023-07-14

**Authors:** Amir Ayali, Gal A. Kaminka

**Affiliations:** ^1^School of Zoology, Sagol School of Neuroscience, Tel Aviv University, Tel-Aviv, Israel; ^2^Department of Computer Science and Gonda Brain Research Center, Bar-Ilan University, Ramat Gan, Israel

**Keywords:** locusts, collective motion, visual perception, bio-inspired, robot collective behavior, vision-based

## Abstract

Swarming or collective motion is ubiquitous in natural systems, and instrumental in many technological applications. Accordingly, research interest in this phenomenon is crossing discipline boundaries. A common major question is that of the intricate interactions between the individual, the group, and the environment. There are, however, major gaps in our understanding of swarming systems, very often due to the theoretical difficulty of relating embodied properties to the physical agents—individual animals or robots. Recently, there has been much progress in exploiting the complementary nature of the two disciplines: biology and robotics. This, unfortunately, is still uncommon in swarm research. Specifically, there are very few examples of joint research programs that investigate multiple biological and synthetic agents concomitantly. Here we present a novel research tool, enabling a unique, tightly integrated, bio-inspired, and robot-assisted study of major questions in swarm collective motion. Utilizing a quintessential model of collective behavior—locust nymphs and our recently developed Nymbots (locust-inspired robots)—we focus on fundamental questions and gaps in the scientific understanding of swarms, providing novel interdisciplinary insights and sharing ideas disciplines. The Nymbot-Locust bio-hybrid swarm enables the investigation of biology hypotheses that would be otherwise difficult, or even impossible to test, and to discover technological insights that might otherwise remain hidden from view.

## 1. Introduction

All swarm systems—natural and robotic—share a common characteristic: No single individual is able to perceive all the others; and no individual directly acts on all the others. A self-organized large-scale (collective-wide) order, nonetheless emerges, despite the inherently localized perception and action of the individuals composing the system. Coordination arises from the complex triadic-interactions between the individual swarm member (its perception, decision-making), the physical surroundings (terrain, topography, obstacles, and threats), and the social environment (peers/conspecifics; other swarm members).

In this short paper we present a perspective on a powerful new research tool for the study of natural and robot swarms: the *Nymbot-Locust bio-hybrid swarm*, that combines multiple robots and animals collectively moving together in a laboratory setting. We believe that the use of the bio-hybrid swarm is conductive to novel research directions, tightly integrating biology and robotics, facilitating our understanding of collective motion, and thereby benefiting both disciplines.

In Section 2 below, we focus on the study of *collective motion in swarms*, a phenomenon that appears ubiquitously in nature (including human societies). We briefly review the theoretical and experimental background in the study of collective motion, from abstract mathematical models, through computer simulations, to animal and robotic model systems, in virtual and physical environments. We follow this with a discussion of the synergistic biology-robotics approach (Section 3) in general, and specifically leading to the *Nymbot-Locust bio-hybrid swarm*. We end (Section 4) with a discussion of future prospects and research directions for investigating swarms, as emerging from this powerful new tool and facilitated by it.

## 2. Swarming and coordinated collective movement

**Figure 1:**
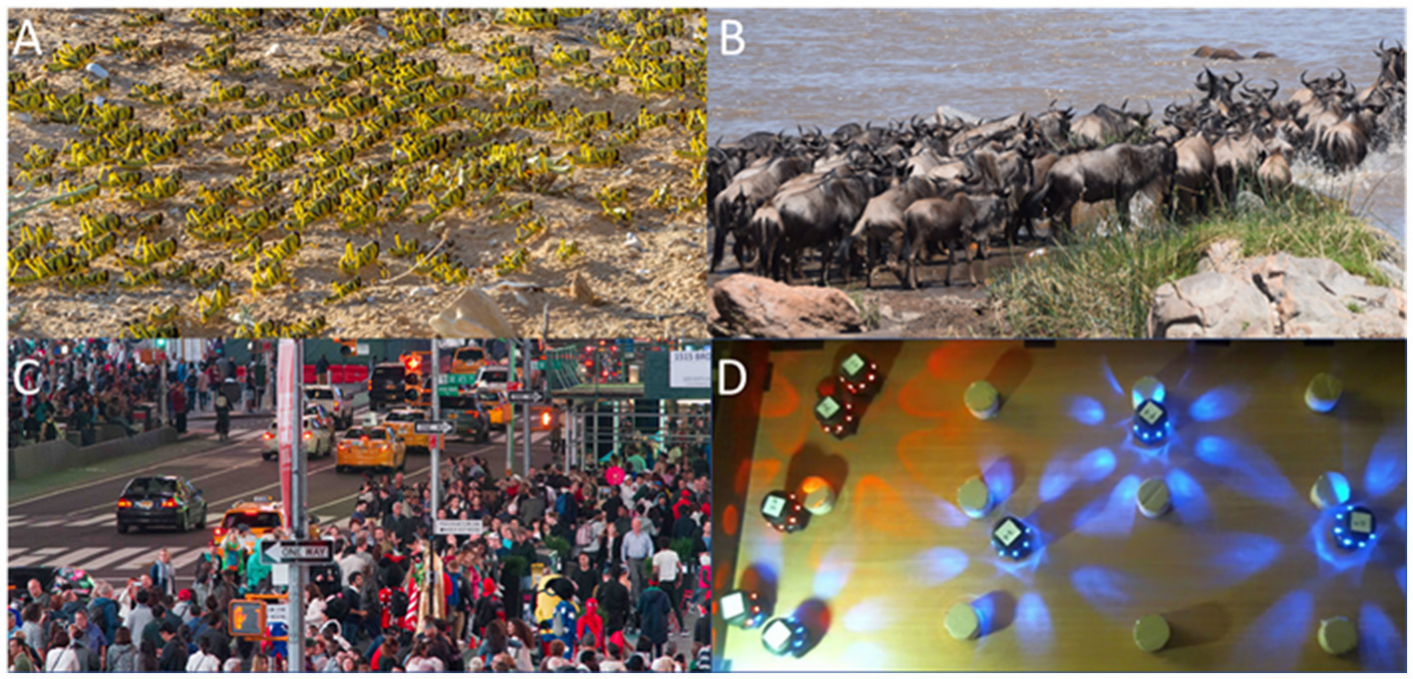
Examples of self-organized collective motion systems. **(A)** A marching swarm of desert locust nymphs, Israel, Negev desert, 2013. **(B)** A herd of Gnu crossing Tanzania's river Mara. **(C)** Pedestrian collective motion. **(D)** A swarm of Krembot robots, negotiating their environment.

Nature presents ample examples of coordinated, collective motion of large swarms of individual organisms: bird flocking, fish schooling, herd stampeding, insect swarming, human pedestrian traffic, and crowd evacuation (Wolff, 1973; Patterson et al., 2007; Moussaïd et al., 2009; Sumpter, 2010; Barnett et al., 2016; Ward and Webster, 2016; see Figure 1). These important natural phenomena have inspired considerable research efforts, ranging from analysis in mathematics, computer science, and physics (Henderson, 1971; Vicsek et al., 1995; Edelstein-Keshet, 2001; Helbing et al., 2001; Giardina, 2008; Vicsek and Zafeiris, 2012), through the development of synthetic swarms in graphics (Reynolds, 1987; Tu and Terzopoulos, 1994) and simulations (Blue and Adler, 2000; Helbing et al., 2001; Daamen and Hoogendoorn, 2003; Toyama et al., 2006; Tissera et al., 2007; Fridman and Kaminka, 2010; Tsai et al., 2011; Kaminka and Fridman, 2018), to robotics (Matari, 1994; Svennebring and Koenig, 2004; Correll and Martinoli, 2009; Kernbach et al., 2010; Mayet et al., 2010; Rubenstein et al., 2012; Brambilla et al., 2013; Giuggioli et al., 2016; LeventBayindir, 2016; Haghighat and Martinoli, 2017; Gauci et al., 2018; Hamann, 2018; Schranz et al., 2020; Dorigo et al., 2021; see Figure 1).

### 2.1. Theoretical studies of collective motion

A wide range of theoretical explanations exists for the emergence of collective motion in animals (see reviews in Edelstein-Keshet, 2001; Giardina, 2008; Eftimie, 2012; Vicsek and Zafeiris, 2012; Escobedo et al., 2020), and many attempts have been made to relate these to simulations and robotic swarms (Reynolds, 1987; Parker, 1993; Matari, 1994; Brutschy et al., 2014; LeventBayindir, 2016; Hamann, 2018; Schranz et al., 2020; Dorigo et al., 2021).

The commonly accepted view is that the behavior of the individual in a swarm—a bird, a fish, an insect, a computer-simulated agent, or a swarming robot—is generated by its repeated assessment of its locally-perceived social and physical environments, and its reaction to these (Vicsek et al., 1995; Vicsek and Zafeiris, 2012).

The theoretical, abstract view of collective motion mostly offers an agent-based perspective (also known as SPP, for self-propelled particle) that prescribes the dynamics of an abstracted individual (the organism's “Umwelt” in biology von Uexküll, 1992). Individual dynamics are computed by modulating the velocity of each individual according to the proximity and velocity of others. For example, one popular family of algorithms for generating collective motion requires every agent to adopt the mean heading of those near it, within a certain range (Giardina, 2008; Vicsek and Zafeiris, 2012). The result, when viewed on a computer screen, can display a striking visual similarity to animal flocking (swarming).

### 2.2. Empirical studies of collective motion

A common and conspicuous drawback of the theoretical abstract models lies, however, in the difficulty of relating them to *embodied* properties of individual animals or robots. The models generally ignore body morphology, size, and kinematics (which constrain responses to obstacles and inter-swarm collisions). Bodily states, such as hunger or energy levels, as well as species-wide perceptual and cognitive capacity (such as the geometry of the visual field or motion prediction capabilities) are also ignored. Moreover, as a result of abstracting the embodied characteristics, individual variances within the swarm are generally also disregarded (Ariel et al., 2022).

Two particular scientific disciplines—biology and robotics—have a shared interest in exploring the gaps in the theoretical understanding of collective swarm motion. Both disciplines investigate *embodied physical agents* and are concerned with kinematics, energy, and mechanical constraints. Such studies, however, are limited by computational power and perceptual capabilities; as well as by the challenges introduced by literal and logical obstacles in the agents' physical environment. Both disciplines are acutely aware of the gaps between abstracted models and physical reality, and have a deep interest in empirical research methods.

#### 2.2.1. Collective motion in biology

Natural models utilized in the study of collective behavior range from bacteria, insects, fish, and birds, to several mammalian species, including humans. Many of these studies are based on empirical observations of collective motion in natural settings (Sumpter, 2010; Ward and Webster, 2016; Papadopoulou et al., 2023), including studies of human collective motion (Herbert-Read et al., 2013; Bierbach et al., 2020). However, organisms that lend themselves to laboratory experiments enable the investigation of well-defined specific questions in a highly controlled manner. Bacteria, for example, constitute an excellent model for laboratory investigation of synchronized collective motion. Indeed, studies of various bacterial species have contributed to a qualitative understanding of the collective motion phenomenon (e.g., Zhang et al., 2010; Beer et al., 2020). Perhaps the most common among collective motion laboratory studies are those utilizing fish, from fish pairwise interactions (e.g., Herbert-Read et al., 2013; Bierbach et al., 2020) to collective alignment in groups (e.g., Harpaz et al., 2021), and more.

Among the organisms that serve as laboratory models for collective motion studies, *locusts*, in particular, have proven to be both useful and successful in generating important insights into the underlying mechanisms (e.g., Ariel and Ayali, 2015; Buhl and Rogers, 2016). Locusts are short-horned grasshoppers belonging to the family Acrididae. The various species of locusts are all characterized by their tendency to form large swarms that can comprise millions of individual insects. These swarms can travel long distances, consuming all the vegetation in their path and causing severe damage to agricultural crop and other plants. The tendency toward establishing collective motion exists in locusts regardless of age. While adult locusts fly, the juvenile forms of locusts—nymphs or hoppers—demonstrate a synchronized collective motion that is known as marching bands (Figure 1): Huge numbers of hoppers will coordinate their behavior and cover large distances in swarms covering vast areas.

As noted, this inherent tendency for swarm collective motion, coupled with the locusts' capacity to demonstrate coordinated behavior even in simple, controlled, laboratory settings (in addition to the ease and low price of breeding and keeping them in the lab), make this insect a canonical experimental model for studying collective motion. Laboratory experiments with locusts enables the control of environmental conditions (such as the general shape of the experimental arena, presence of obstacles or roughness of the terrain) as well as focusing on particular aspects of the social environment (e.g., breeding density of the locusts, density of the swarm, and group variability). In addition, because vision is considered the primary sensory modality used by hoppers within the locust swarm (Ariel et al., 2014; Ariel and Ayali, 2015; Bleichman et al., 2023), analysis and simulation of their perception is made relatively viable (in comparison to auditory or olfactory perception).

Laboratory experiments with live animals—locusts or other—still present a major challenge, however, in regard to controlling and manipulating the specific social environment of the individual, i.e., the peers visible to it and moving around it; and, moreover, the nature of the intra-swarm interactions. Namely: when investigating the behavioral response of the individual, we cannot control the behavior of its peers, or make them go in one direction or the other, in order to observe the consequent decision of the individual. Fully-controlled swarm-wide experiments still remain beyond the reach of this methodology.

#### 2.2.2. Collective motion in robotics

Roboticists have been studying collective motion in *multi-robot systems* for many years. Much of this research has focused on non-swarm settings, in which the robots are centrally-controlled, or their coordination is tightly managed via group-wide (“global”) communication among them, in order to achieve the explicit joint goals of the group and to carry out its tasks collaboratively.

There is, however, a separate and distinguished body of research into multi-robot systems that focuses on robotic swarms (Matari, 1994; Kernbach et al., 2010; Mayet et al., 2010; Rubenstein et al., 2012; Brambilla et al., 2013; LeventBayindir, 2016; Haghighat and Martinoli, 2017; Gauci et al., 2018; Hamann, 2018; Schranz et al., 2020; Dorigo et al., 2021), very much in parallel to the above described biological work. It focuses on highly-localized perception and action, leading to numerous local interactions and an emergent order. The use of robot swarms has been demonstrated for a variety of tasks: foraging (Rosenfeld et al., 2008; Kaminka et al., 2010; Douchan et al., 2019), navigation (Mayet et al., 2010), area coverage (Svennebring and Koenig, 2004; Correll and Martinoli, 2009; Giuggioli et al., 2016), and many more (Rubenstein et al., 2012; Brutschy et al., 2014; Werfel et al., 2014; Haghighat and Martinoli, 2017; Gauci et al., 2018). Matari (1994), for example, reported on collective movement—flocking—by applying the theoretical models presented by Reynolds (1987), using a range of sensors to estimate distances to peers, and their velocities. Similar studies have been able to replicate and further improve this approach.

Despite the well-recognized role of visual perception for collective vision in the natural world, it has been challenging to achieve robot collective motion using visual perception alone, without direct range measurements (but see Moshtagh et al., 2009; Serres and Ruffier, 2017; Berlinger et al., 2021 for a few exceptions). Clearly, swarm-robotics could benefit greatly from a better understanding of natural swarms, and specifically, from insights into the question of how visual perception is used in nature for coordinated swarm movement.

### 2.3. Synergistic bio-robotic studies of collective motion: the gap

Shared interests of biologists and roboticists have led to advances in several areas of research other than that of swarms and collective motion. Over the years there have been studies that exploited the complementary nature of the two disciplines: *bio-inspired robotics* traditionally draws inspiration from nature and applies it to technological advances (Bonabeau et al., 1999; Kernbach et al., 2010; Werfel et al., 2014); while *robotics-assisted biology* utilizes robots as a way to create dynamically-controlled conditions, enabling the testing of biology hypotheses that could otherwise not be tested (Balch et al., 2006; Gribovskiy et al., 2010; Krause et al., 2011; Bonnet et al., 2012; Porfiri et al., 2019; Horsevad et al., 2022; see Romano and Stefanini, 2021a for a recent collection).

One would have expected to see numerous published empirical studies of swarms, conducted jointly by biologists and roboticists, whose shared interests are so compatibly aligned. Unfortunately, such studies are rare, and typically use only a single robot (or very few) to interact with a group of animals. Asadpour et al. (2006) and Halloy et al. (2007) used a human-controlled robot to investigate the role of pheromones in cockroach group shelter selection. Romano et al. (2020) used beetle-robot interactions to investigate lateralization effects on behavior. da Silva Guerra et al. (2010) investigated cricket fighting dynamics in response to a miniature robot's behaviors. Polverino et al. (2012) and Butail et al. (2013) reported on zebrafish individual and shoaling responses to a single robotic fish. There are many other examples, (e.g., Romano et al., 2017, 2019; Romano and Stefanini, 2021b, 2022; Worm et al., 2021). Single-robot use has reached commercial applications: a human-controlled ornithopter drone with falcon appearance is available to repel bird flocks from dangerous areas (Robird; Folkertsma et al., 2017).

Experiments involving multiple robots and many swarming animals, coordinating their movement and co-interacting with their environment are, however, greatly lacking. No doubt, this is due to the difficulty of controlling many individual robots moving collectively, to study their *controlled* collective motion-induced effects on the individual animal and its peers.

## 3. The Nymbot-Locust bio-hybrid swarm

Motivated by the above-noted gap, and the mutual needs of robotics and biology studies of swarms, we have been developing the *Nymbot-Locust bio-hybrid swarm*, a novel empirical research platform that enables joint studies of natural and artificial swarms. The bio-hybrid swarm platform combines multiple live locusts with multiple specially-designed robots (called *Nymbots*, for Nymph-Robot), enabling controlled experiments in laboratory settings. The bio-hybrid swarm constitutes a unique tool for conducting tightly-integrated, synergistic, bio-inspired and robot-assisted swarm research; *a “super-collider” for swarm researchers*, and one that can facilitate discoveries in both biology and robotics.

The Nymbots have been designed and developed especially for bio-hybrid swarm research. While locusts use multiple modalities to communicate (visual, tactile, and semiochemical), it is well-accepted that vision is the dominant sensorial modality in their collective motion, especially preceding adult emergance. Therefore, their design is heavily constrained not only by technological feasibility, but also by the need for them to be compatible with locust hoppers in size, shape, and overall gross behavior as perceived by the animals.

**Figure 2:**
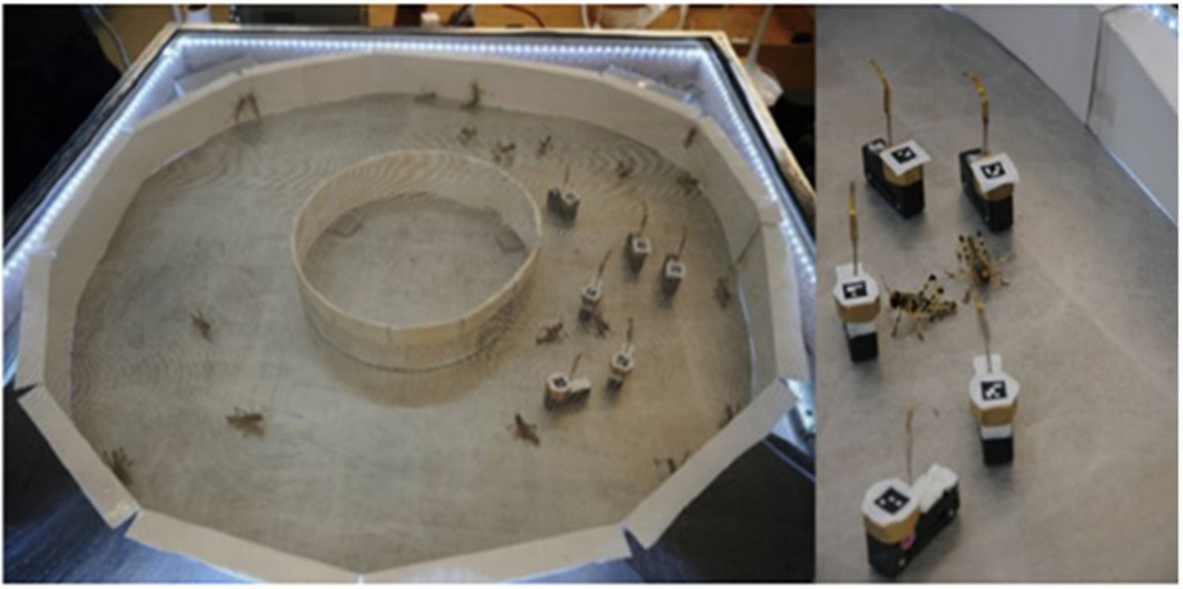
The Nymbot-Locust bio-hybrid swarm: A group of last-instar gregarious desert locust nymphs interacting with the miniature robotic devices, demonstrating coordinated collective motion under controlled laboratory conditions. The metal mesh covered arena **(left)**; close-up on several Nymbots and friends **(right)**.

A key design requirement was to enable the robots to function continuously for several hours, as an individual locust experiment can often take 2–3 h. Given the restrictions on the size of the robots (~5 cm in length, 1.2 cm in width, and 1.2 cm in height), this requirement proved to be very challenging. The robots receive continuous DC power from the arena's metal floor and metal mesh ceiling, which is placed at a height of 6 cm, a sufficient height to prevent the live insects from being electrocuted by touching both floor and ceiling simultaneously. The metal mesh ceiling enables an overhead camera to track both robots and animals using visual fiducials (Figure 2). While in the future the goal is to achieve fully distributed control, a separate central computer currently autonomously controls the robots' behavior in response to their physical and social surroundings. The software and hardware designs of the Nymbots are open-source and available to any interested party.

The basic experimental arena used to date is rectangular, with a circular wall inside it, creating a ring-shaped (donut) corridor, in which the locusts and Nymbots swarm together. The Nymbots and nymphs (last-instar gregarious desert locusts) are introduced into the arena at sufficiently high densities to enable the formation of synchronized movement by the insects (10–50 in most experiments). The robots are clearly accepted by the locusts as they move in the arena (Figure 2). We note in particular that the familiar pause-and-go pattern of movement that is characteristic of locust swarms (Ariel et al., 2014) is evident in the locust responses to the robots' motion, despite the continuous nature of the latter. While this simple arena has already generated important insights regarding the convergence of individuals to swarm together under different densities and rearing conditions, it is expected to enable additional in-depth testing of decision-making toward collective motion, as well as during it. We are currently mainly focusing on establishing dynamical control of the visual stimuli available to the locust, while conducting a preliminary analysis of movement coordination under visual perception alone.

## 4. Discussion

This is a perspective paper, reporting on preliminary results from the trenches, from the very forefront of bio-hybrid swarm research. We have very briefly presented here the case for the approach and design of the robots, the swarm arena, and the currently on-going experiments. Fifty Nymbots have been built to date and are being deployed as we continue our research; and a 3D physics-based simulator has been developed to accelerate the development of robot controllers.

The underlying conceptual cornerstone of the bio-hybrid swarm as a research tool lies in that it enables varying the ratio of locusts to Nymbots. This conduces to shifting the focus of experiments from testing hypotheses on *computational* decision-making, to testing hypotheses on *natural* decision-making: At one extreme, a few locusts introduced into a swarm of many robots—whose behavior and appearance are appropriately controlled— enables testing hypotheses on the individual locust decision-making in response to visual and motion stimuli generated by their peers. At the other extreme, a few Nymbots introduced into a swarm of many locusts enables determination of whether certain robot control processes, or perception processes, are more effective than others. Moreover, if specific algorithms generate behavior by the Nymbots that is better accepted by the locusts, we may also be able to construct algorithms that can serve as faithful models of locust decision-making. Other locust-to-Nymbot ratios have enabled us to test hypotheses regarding the effects of the individual decision-making processes on the group. For the locusts, we use both individual and group quantitative measures of behavior to test the effects of manipulations. The robots can be directly queried for their internal states.

One important open question that we are currently pursuing in our research, for example, is related to the underlying perceptual mechanisms that allow the locust to coordinate their movements in the swarm. Elsewhere (Krongauz et al., 2023), we have shown that robust collective movement can be generated using vision alone, if the perception process is able to visually recognize peers (conspecifics) and to differentiate them from other moving elements in the visual field. However, this finding would seem to contradict the results from experiments with a single locust, that responded favorably to visual stimuli of *randomly-placed dots* (rather than conspecific images) moving in the same direction (Bleichman et al., 2023). We expect our Nymbot-Locust bio-hybrid swarm will facilitate our research and understanding of this and other important questions in future collective motion research.

## Data availability statement

The original contributions presented in the study are included in the article/supplementary material, further inquiries can be directed to the corresponding authors.

## Author contributions

All authors jointly conceived and wrote the paper. All authors contributed to the article and approved the submitted version.
